# Comparing the survival rate of juvenile Chinook salmon migrating through hydropower systems using injectable and surgical acoustic transmitters

**DOI:** 10.1038/srep42999

**Published:** 2017-02-21

**Authors:** Z. D. Deng, J. J. Martinez, H. Li, R. A. Harnish, C. M. Woodley, J. A. Hughes, X. Li, T. Fu, J. Lu, G. A. McMichael, M. A. Weiland, M. B. Eppard, J. R. Skalski, R. L. Townsend

**Affiliations:** 1 Pacific Northwest National Laboratory, P.O. Box 999, Richland, WA 99332, USA; 2 U.S. Army Corps of Engineers, 333 SW First Avenue, Portland, OR 97204, USA; 3 School of Aquatic and Fishery Sciences, University of Washington, 1325 Fourth Avenue, Suite 1820, Seattle, WA 98101, USA

## Abstract

Acoustic telemetry is one of the primary technologies for studying the behavior and survival of fishes throughout the world. The size and performance of the transmitter are key limiting factors. The newly developed injectable transmitter is the first acoustic transmitter that can be implanted via injection instead of surgery. A two-part field study was conducted to evaluate the performance of the injectable transmitter and its effect on the survival of implanted fish. The injectable transmitter performed well and similarly to the proceeding generation of commercially-available JSATS transmitters tested concurrently. Snake River subyearling Chinook salmon smolts implanted with the injectable transmitter had a higher survival probability from release to each of eleven downstream detection arrays, because reach-specific survival estimates were significantly higher for the injectable group in three of the eleven reaches examined. Overall, the injectable group had a 0.263 (SE = 0.017) survival probability over the entire 500 km study area compared to 0.199 (0.012) for the surgically implanted group. The reduction in size and ability to implant the new transmitter via injection has reduced the tag or tagging effect bias associated with studying small fishes. The information gathered with this new technology is helping to evaluate the impacts of dams on fishes.

Acoustic telemetry is commonly used throughout the world for studying the behavior and survival of fishes. A primary assumption of behavior and survival studies necessitates that fish used in the study are representative of the wild population of interest with regard to physical characteristics (e.g., size), behavior, and survival^1^,^2^. Although substantial progress has been made to minimize the size of transmitters^3^,^4^,^5^ and improve transmitter attachment procedures^6^,^7^,^8^ in order to meet this assumption, tag or tagging effects may persist^9^, which can lead to biased study results.

The potential for a tag effect, whereby the presence of the transmitter negatively influences the implanted fish’s performance, is typically measured in terms of tag burden (i.e., the weight of the transmitter relative to the weight of the fish)^10^. Numerous researchers have demonstrated the swimming ability, predator avoidance, growth, tag retention, or survival of implanted fish may be compromised if the tag burden is large (e.g., refs 11, 12). Evolution in micro-battery technology in recent years^13^,^14^ has resulted in the production of smaller acoustic transmitters. In addition to enabling the implantation in smaller fish, these advancements have also enabled researchers to reduce the tag burden experienced by implanted fish, thereby reducing the tag effect.

Despite reductions in transmitter size, currently used transmitter attachment methods may cause a tagging effect, whereby the attachment procedure itself negatively influences fish performance^9^. Of the three most commonly used methods for attaching acoustic transmitters to fish (surgical, gastric, and external), surgical implantation is thought to be the best attachment method for longer term telemetry studies^15^. Internal implantation of the transmitter provides protection from environmental entanglement and eliminates drag forces during swimming, which are complications of external attachment, while also having less of an effect than gastric insertion on the tagged fish’s feeding ability^16^. However, there are disadvantages to surgical implantation. Due to the invasive and intricate nature of the surgical process, implantation may require deeper anesthesia, greater handling of study fish, and more time to execute than the other attachment methods. Additionally, the use of sutures may contribute to inflammation and infection if the sutures remain in fish longer than needed^17^,^18^. Therefore, reducing the amount of time and handling required to implant fish as well as the amount of suture material may help to decrease the tagging effect.

Recently, a Juvenile Salmon Acoustic Telemetry System (JSATS) acoustic micro-transmitter was developed that can be implanted in fish by injection instead of surgery^13^,^19^,^20^. Due to the reduction in handling and shorter anesthesia and implantation times, and the lack of a need for sutures, transmitter injections may have less of a tagging effect than surgical implantations^19^. Additionally, the injectable transmitter weighs 30% less than other available acoustic micro-transmitters. The smaller size of the injectable transmitter results in lower tag burdens, which may help to reduce the tag effect. We conducted a field study to evaluate the performance of the newly developed injectable transmitter and its effect on the survival of implanted fish. The in-river survival of fish implanted with the injectable transmitter was compared to concurrent releases of fish that were surgically implanted with currently available single-battery JSATS transmitters and released as part of a multi-dam performance standard evaluation (PSE) study.

## Methods

This section describes the transmitters and study methods used for the in-river survival comparison. The in-river survival evaluation compared the survival and detection probabilities of two groups of subyearling Chinook salmon during their emigration through 500 km of the Snake and Columbia rivers. The two groups consisted of the treatment group, which was implanted with the injectable transmitter, and the control group, which was surgically implanted with a currently available single-battery JSATS transmitter. For all comparisons, the methods used were identical among transmitter types, except for the tagging procedure used to implant the different transmitter types for the in-river survival comparison.

### Acoustic transmitters

**Figure 1:**
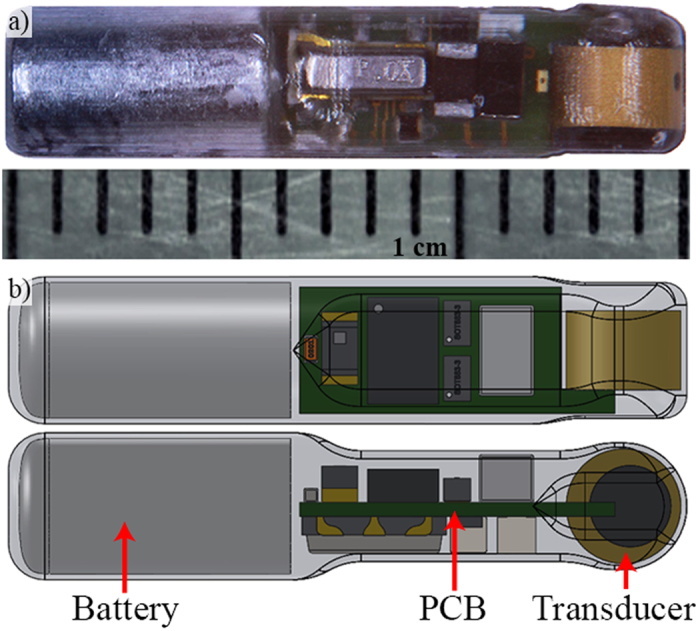
Actual injectable transmitter shown with a scale (**a**), and a Computer-Aided Design model of the injectable transmitter showing the actual shape of the transmitter (**b**).

The injectable transmitter used in this study (Fig. 1) were manufactured by engineers at the Pacific Northwest National Laboratory (PNNL). Each injectable transmitter (model micro V1) measured 15 mm in length, had a diameter of 3.35 mm, a volume of 0.111 mL, and weighed 0.216 g in air and 0.105 g in water. The general shape of the transmitter is cylindrical; excess epoxy was eliminated to reduce the weight and epoxy surrounding the transducer element was minimized. Injectable transmitters used for the comparison had a nominal transmission rate of 1 pulse every 4.2 s and a nominal source level of 155 dB re 1 μPA at 1 m. Nominal tag life of the injectable transmitters was expected to be about 28 d with a 4.2 s ping rate. A more recent version of the injectable transmitters (model micro V2) has a tag life that is over four times longer than the version used in this study^20^.

The single-battery JSATS transmitters surgically implanted in fish of the control group for the in-river survival comparison were manufactured by Advanced Telemetry Systems, Inc. (ATS; Isanti, Minnesota). Each transmitter (model number SS300) was 10.79 mm long, 5.26 mm wide, 3.65 mm high, and weighed 0.346 g in air. The transmitters had a nominal transmission rate of 1 pulse every 4.2 s and a nominal source level of 155 dB re 1 μPA at 1 m. Nominal tag life was expected to be about 45 d with a 4.2 s PRI.

### In-river survival comparison

#### Fish source

The subyearling Chinook salmon (*Oncorhynchus tshawytscha*) tagged for this study were obtained from the Lower Monumental Dam (LMN) juvenile bypass system (JBS). Fish implanted with injectable transmitters were selected using the same criteria used to select the fish of the surgically implanted control group, which were tagged with a single-battery JSATS transmitter as part of the PSE study. Fish selected for the current study were maintained in holding tanks for 18 to 30 h prior to surgery. The size of fish selected for implantation with the injectable transmitter was similar to that of fish in the surgically implanted group (Supplementary Table S5). Holding conditions and all experimental procedures were approved by and carried out in accordance with guidelines of the Institutional Animal Care and Use Committee (IACUC) of PNNL.

#### Tagging Procedure

One to three subyearling Chinook salmon were netted at a time from a holding tank and placed into a container and anesthetized using tricaine methanesulfonate (i.e., MS-222) to Stage 4 as described by Summerfelt and Smith^21^. Anesthetized fish were then transferred to a data collection station where information regarding the physical attributes of the fish and relevant tagging information were recorded. For fish of the treatment group, a disinfected PIT tag and an injectable transmitter assigned to that fish were inserted into a sterilized 8-gauge stainless steel hypodermic needle while the fish was at the data station. The injectable transmitter was first placed into the needle battery-end first. The PIT tag was then inserted into the needle below the acoustic transmitter. A sanitized plastic cap was then put over each end of the needle to keep both tags in place. Once both tags were placed into the needle it was handed to the surgeon as the fish arrived at the tagging station.

The fish was placed on the surgery table and given a maintenance anesthetic dose. The exact dose was controlled by the surgeon during the procedure by mixing river water and the maintenance anesthetic water to maintain Stage 4 anesthesia. The surgeon then removed the top cap of the needle and attached the syringe-style implanter to the needle. Once attached, the surgeon removed the second cap from the needle and inserted the needle into the skin of the fish, with bevel up, at an angle of 30–40 degrees (Supplementary Fig. S4). The insertion point was at the end of the pectoral fins, offset from the ventral midline. Once the abdominal tissue was cut, the needle/syringe combination was twisted 90° (note that the surgeon watches the bevel to determine rotation) and the PIT and acoustic tags were inserted. Care was taken to ensure the needle did not enter the body cavity beyond the 50% bevel of the needle, which is approximately 7 mm from tip. Once the tags were injected, the needle was removed and the fish was placed into a container for photographs. The insertion site did not require suturing. Once the injection was completed, each fish was then placed into a chute with a continuous supply of fresh river water that emptied into 24.6-L transport buckets. A maximum of six fish were put into each transport bucket.

The anesthetization and data management procedures used for fish in the surgically implanted group were similar to those described above for the fish implanted with injectable transmitters. However, as mentioned previously, the transmitter implantation procedure differed between the two groups and different taggers were used. The surgical implantations were performed as described by Deters *et al*.^18^. The incision was closed using two simple, interrupted sutures tied with reinforced square knots that were made with one wrap on each of four throws.

#### Release Procedure

The same methods were used to release both the fish implanted with the injectable transmitters and the surgically implanted fish. The fish were transported in separate insulated totes by the same truck from the tagging site at LMN to the release site located at river kilometer (rkm) 655 (as measured from the mouth of the Columbia River; see Supplementary Fig. S5 for a map of the sites). Releases occurred for 11 consecutive days (between 22 June and 2 July 2013) and were staggered between day and night to match the PSE study design.

#### Acoustic Signal Detection and Processing

Acoustic transmissions from tagged fish were detected and decoded by autonomous and cabled acoustic telemetry receivers. Autonomous receivers were deployed prior to the release of tagged fish using the methods described by Titzler *et al*.^22^. Autonomous receiver arrays were deployed at 9 different transects in the Snake River between the forebay of Little Goose Dam (LGS; rkm 636) and the Snake River mouth (rkm 522) and at 3 locations in the Columbia River between the forebay of Bonneville Dam (rkm 236) and Kalama, Washington (rkm 126). Cabled acoustic telemetry receiving systems^23^,^24^ were deployed on the dam faces at LGS and LMN. The hydrophones of the cabled systems were deployed as described by Li *et al*.^25^. Detections of tagged fish on both the autonomous receivers and cabled systems were used to calculate travel times and estimate survival probability. Details on the data filtering and processing can be found in the Supplemental Information.

#### Survival and Detection Probability Estimation

Detection and survival probabilities were estimated using the single-release/recapture model first presented by Cormack^26^, Jolly^1^, and Seber^2^, and later by Skalski *et al*.^27^. Valid tag code detections were used to construct detection histories for each fish at all survival arrays. Detection and cumulative survival probabilities were estimated for all fish of each transmitter group from the release location at Central Ferry (rkm 655) to each downstream detection array (Supplementary Table S6). The single-release/recapture model was also used to estimate survival within each reach located between detection arrays down to the Knapp, Washington array at rkm 152. Reach-specific survival was estimated for all fish of a transmitter type that were detected by the array that marked the upstream boundary of the reach.

For each survival estimate, detections on all downstream arrays were pooled to develop the detection history of the secondary array. The autonomous receiver array at Kalama (rkm 126) was used as the secondary array to estimate the detection and survival probability of the Knapp (rkm 152) array. Because no opportunities for detection existed downstream of the Kalama array, survival and detection probability could not be estimated for this array. Detection histories were uploaded into the program SURPH (version 3.5.2; http://www.cbr.washington.edu/analysis/apps/surph) to calculate survival and detection probabilities. Likelihood ratio tests (*α* = 0.05) were used to evaluate the likelihood of equal versus unequal survival and detection probability between transmitter/implantation types.

## Results

### In-river Survival Comparison

The probability of detection was very high for both the injectable and single-battery JSATS transmitters at all autonomous and cabled receiver arrays. The majority of arrays had detection probabilities of 1.0 and all arrays exceeded 0.99 detection probability for both transmitter types. We observed no significant differences in detection probability between the two transmitter types at any of the arrays (*χ*^2^ ≤ 1.253; *P* ≥ 0.263).

Travel times were similar between subyearling Chinook salmon implanted with the injectable transmitter and those surgically implanted with the single-battery JSATS transmitter. The large majority of fish from both groups had exited the Snake River sometime around mid-to-late July, and exited the study area in the Columbia River by the end of July.

**Figure 2:**
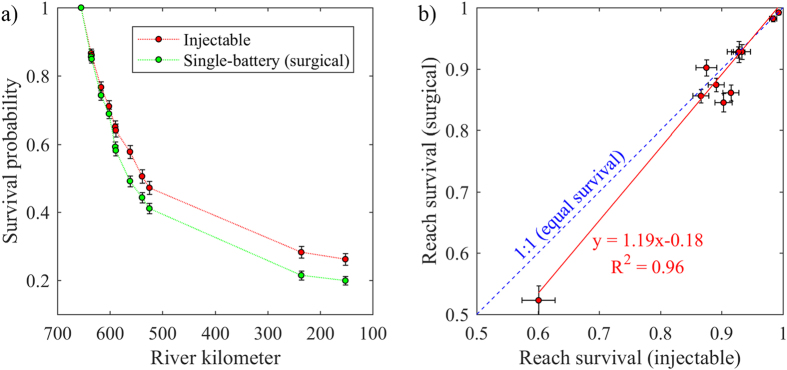
Survival estimate comparison between the subyearling Chinook salmon implanted with the injectable transmitter and the control group which were surgically implanted with the single-battery JSATS transmitter. (**a**) Cumulative survival probability from the release site (rkm 655) to each downstream detection array with error bars denoting the standard errors; (**b**) Reach specific survival probabilities with horizontal and vertical error bars that denote the corresponding standard errors. The dashed blue line represents equal survival between the groups and the red line is a linear regression through the data points.

Subyearling Chinook salmon implanted with the injectable transmitter had a higher probability of survival from release to each downstream detection array than those surgically implanted with the single-battery JSATS transmitter (Fig. 2a). The difference in cumulative survival (from release) between the two transmitter groups first became significant at the detection array located in the LMN forebay (rkm 589; *χ*^2^ = 5.765; *P* = 0.016). Fish implanted with the injectable transmitter had a 0.650 (SE = 0.018) probability of survival from release to the LMN forebay compared to 0.592 (0.015) for fish surgically implanted with the single-battery JSATS transmitter. The greatest statistically significant difference in cumulative survival between transmitter types was observed 93 km downstream from the release location at the rkm 562 array where fish implanted with the injectable transmitter had a 0.578 (0.019) survival probability compared to 0.492 (0.016) for the surgically implanted group (*P* = 0.001). Overall, subyearling Chinook salmon implanted with the injectable transmitter had a 0.263 (0.017) probability of survival over the entire 500 km study area (from release to the rkm 152 array) compared to 0.199 (0.012) for fish of the surgically implanted group (*P* = 0.002).

The injectable group had equal or higher reach-specific survival estimates than the surgically implanted group for all reach’s except for the reach between rkm 562 and rkm 539 (Fig. 2b) where the surgically implanted group was higher, although the difference was not significant (*P* = 0.213; Supplementary Table S7). The first significant difference in reach-specific survival between the two groups was observed in the LMN forebay between rkm 602 and rkm 590 where the injectable group had a survival probability higher than the surgically implanted group (*P* = 0.004). The injectable group also had a significantly higher probability of survival than the surgically implanted group in the reach that included passage through LMN, from the cabled array deployed on the face of LMN (rkm 589) to the next downstream array (rkm 562; *P* = 0.005). For the longest reach examined, which extended from the array near the mouth of the Snake River (rkm 525) to the forebay of Bonneville Dam (rkm 236) and included passage through McNary, John Day, and The Dalles dams, the injectable group had a significantly (*P* = 0.032) higher probability of survival than the surgically implanted group. This reach also corresponded to the largest reach-specific survival difference obtained.

## Discussion

Fish implanted with the injectable transmitter had a higher probability of survival from release to each downstream detection array than the fish that were surgically implanted with the 2013 single-battery JSATS transmitter, with the difference in cumulative survival becoming significant 65 rkm from the release site and maintaining significance to the last detection array 503 rkm from the release site. In addition, reach-specific survival estimates were significantly higher for the injectable group in three of the eleven reaches examined in this study. The largest significant difference in reach-specific survival corresponded to the physically longest migration segment that spanned 289 rkm and included passage through three separate dams. It appears the reduction in transmitter size and the use of the injection method may have reduced the tag and tagging effect, respectively, compared to the larger transmitter that was surgically implanted. In addition, the injectable transmitter performed extremely well and similarly to the other JSATS transmitters tested in terms of transmitter performance in controlled laboratory and field tests (i.e., detection probability, detection efficiency, tracking efficiency, signal-to-noise ratio, and 3D tracking).

The availability of a smaller acoustic transmitter that can be implanted by injection represents a substantial contribution to the fisheries research community. The reduction in transmitter size may enable smaller fish to be implanted, which provides researchers with the opportunity to better represent the full size range of their population of interest and the ability to study populations, life stages, or species that consist of individuals too small to implant with other available transmitters. In past studies of reservoir, dam passage, and estuarine survival of juvenile salmonids in the Columbia River only smolts that measured ≥95 mm FL were implanted with acoustic transmitters to minimize tag effects (e.g., refs 28, 29). This restriction precluded tagging certain stocks and populations and limited the representativeness of studies that were conducted to the portion of the population larger than this size limit. The availability of the smaller injectable transmitter may enable additional stocks, populations, and life stages of interest to be studied by allowing for the implantation of fish <95 mm FL.

In a laboratory study the 30 day survival of acoustic-tagged juvenile Chinook salmon (*Oncorhynchus tshawytscha*) was not affected until the tag burden exceeded 6.7%^10^. Use of this tag burden “rule” would allow researchers to implant fish as small as 3.2 g with the injectable transmitter without negatively affecting survival. At some point along the fish size-to-transmitter size spectrum, tag volume becomes a more important predictor of fish performance than tag weight^30^,^31^. A juvenile salmon that weighs 3.2 g is unlikely to have sufficient space in its body cavity for the injectable transmitter without putting pressure on internal organs, which could decrease stomach capacity and reduce growth, or provoke other complications^30^,^31^. Research is ongoing to identify the minimum size of juvenile salmonids that can be implanted with the injectable transmitter without affecting fish performance.

The smaller injectable transmitter also provides a reduction in tag burden relative to currently available acoustic transmitters. Previous studies have shown that swimming performance, growth, and survival of implanted fish decline with increasing tag burden^10^,^32^,^33^. We would expect the injectable transmitter to have less of an effect on fish swimming, growth, and survival than all other commercially available acoustic transmitters due to the smaller mass of the injectable transmitter and the corresponding reduction in tag burden. The smaller volume of the injectable transmitter may also reduce biases associated with the presence of the injectable transmitter relative to other transmitters. Fish implanted with transmitters compensate for the additional mass by adding volume to their swim bladders^34^,^35^,^36^. The presence of the transmitter can limit the extent to which the swim bladder can expand, with larger-volume transmitters being more restrictive of normal swim bladder function than smaller transmitters. Reductions in the volume to which the swim bladder can expand limit the range of depths at which tagged fish can achieve neutral buoyancy^37^, thereby altering their behavior, and potentially, their survival.

Transmitter mass and volume are particularly important in studies conducted to evaluate the survival of juvenile salmonids passing through hydropower facilities. During passage through hydroturbines, fish undergo rapid decompression, causing gas in the swim bladder to expand^37^. The presence of a transmitter in the body cavity may prevent the swim bladder from expanding to the size required to compensate for the rapid decompression, causing barotrauma (e.g., compression-related injuries). This was cited as the potential causal mechanism that explained the higher probability of mortal injury of tagged fish in a study that subjected tagged and untagged juvenile Chinook salmon (*Oncorhynchus tshawytscha*) to rapid decompression^37^. We would expect higher turbine passage survival and a reduction in the tag effect bias associated with turbine passage for fish implanted with the injectable transmitter compared to other available acoustic transmitters. In the current study, the group of fish implanted with the injectable transmitter had a significantly higher probability of survival in the river reach that included passage through LMN and in the reach that included passage through McNary, John Day, and The Dalles dams. However, survival of the injectable group was only slightly higher than, or similar to, the surgically implanted group in reaches that included passage through LGS, Ice Harbor, and Bonneville dams. Any differences in turbine passage survival between the transmitter/implantation groups was likely masked in the reach survival estimates due to the low rates of turbine passage that are typical of these dams (i.e., <10%).

Other studies that have made similar comparisons of transmitter size and implantation methods have found that fish injected with a smaller transmitter experienced higher survival than those surgically implanted with a larger transmitter. A combined laboratory and field study was conducted over multiple years (2007 and 2008) to evaluate the effects of surgically implanted JSATS acoustic transmitters into Snake River Chinook salmon juveniles (*Oncorhynchus tshawytscha*)^3^,^9^. The study compared the performance of fish surgically implanted with a JSATS acoustic transmitter and a 0.1 g PIT tag (AT+PIT) to similarly-sized fish that were only injected with a 0.1 g PIT tag (PIT-only). Although the JSATS transmitters used in the study were quite a bit larger than those that are currently available, weighing between 0.42 and 0.66 g, the 2007/2008 study provides valuable insight into the causal mechanisms behind the survival differences observed in the current study.

Results from the field study conducted in 2007 and 2008 indicated that the in-river survival of the AT+PIT and PIT-only groups was similar for larger (≥95 mm FL), spring-migrating yearling Chinook salmon (*Oncorhynchus tshawytscha*) to detection sites located within 225 rkm of release (i.e., Lower Granite Dam to McNary Dam)^3^,^9^. However, the PIT-only group of yearling Chinook salmon had a significantly higher probability of survival than the AT+PIT group to detection sites located farther downstream (i.e., John Day and Bonneville dams). Subsamples of tagged fish collected in the JBS of McNary and Bonneville dams revealed that the surgically implanted AT+PIT group had more inflammation, higher rates of chronic peritonitis and internal adhesions at the incision site, and poorer apposition of the implantation site than the injected PIT-only group.

The survival of AT+PIT and PIT-only groups was only compared for the smaller (≥85 mm FL), summer-migrating subyearling Chinook salmon (*Oncorhynchus tshawytscha*) in 2007, which was a similar water year to 2013 (refer to Supplemental Information for discussion of river environment)^38^,^39^,^40^,^41^,^42^,^43^,^44^,^45^,^46^,^47^,^48^,^49^, characterized by low discharges and high water temperatures in the Snake and Columbia rivers. The PIT-only group of subyearling Chinook salmon had a significantly higher probability of in-river survival than the AT+PIT group between release and the first detection site, which was located within 60 km (about 5 days) of release (i.e., Lower Granite Dam to LGS). The increase in differential survival between the two groups coincided with increases in river temperature.

The results from the 2007/2008 study indicate the difference in survival observed in 2013 between subyearling Chinook salmon implanted with the injectable transmitter and those surgically implanted with the single-battery acoustic transmitter may have been caused by higher rates of infection experienced by the surgically implanted group. The presence of sutures on fish that were surgically implanted may have acted as an attachment site for pathogens, such as fungi or bacteria. Infection rates may have been particularly high in 2013 due to the warm water temperatures experienced in the Snake and Columbia rivers during the summer outmigration period. We hypothesize that the implantation method, rather than the size difference between the transmitters, had the greatest effect on the observed survival difference between the two groups.

Acoustic telemetry continues to be an extremely useful tool for researchers interested in studying the behavior and survival of small fishes. The reduction in size and ability to implant the new transmitter via injection has further reduced the tag or tagging effect bias associated with studying small fishes. Further advancements in battery technology have been made since the completion of this study, enabling the development of an injectable transmitter that has a nominal tag life of 129 days with a PRI of 3 seconds, while maintaining the same small size. The longer tag life of this transmitter further increases the utility of the injectable transmitter by enabling researchers to monitor the movements and survival of tagged fish over a longer period of time. In the Columbia River Basin, the additional tag life provides the opportunity to better understand the behavior and survival of salmonid smolts that don’t emigrate from the river within the typical outmigration season, instead rearing in the reservoirs or estuary for an extended period. The longer tag life also enables researchers to track the migration of tagged smolts along the continental shelf of the Pacific Ocean over greater distances than was previously possible^1^. The information gathered will help better understand the effects dams have on fish, leading to more environmentally sustainable energy systems. The ultimate goal of the transmitter development is to produce a transmitter that (1) produces a reliable and repeatable individually identifiable transmission that can be detected and decoded from a considerable distance; (2) has no effect on implanted individuals; and (3) meets the needs of researchers in terms of battery life. This injectable transmitter represents substantial progress towards that goal.

## Additional Information

**How to cite this article**: Deng, Z. D. *et al*. Comparing the survival rate of juvenile Chinook salmon migrating through hydropower systems using injectable and surgical acoustic transmitters. *Sci. Rep.* **7**, 42999; doi: 10.1038/srep42999 (2017).

**Publisher's note:** Springer Nature remains neutral with regard to jurisdictional claims in published maps and institutional affiliations.

## Supplementary Material

Supplementary Information
