# Supplementary material for: Comparing the survival rate of juvenile Chinook salmon migrating through hydropower systems using injectable and surgical acoustic transmitters
Source: Sci Rep. 2017 Feb 21;7:42999. doi: 10.1038/srep42999 (PMC5318953; doi:10.1038/srep42999)
Supplement: Supplementary Information [file srep42999-s1.doc]

Comparing the survival rate of juvenile Chinook salmon migrating through hydropower systems using injectable and surgical acoustic transmitters

ZD Deng, JJ Martinez, H Li, RA Harnish, CM Woodley, JA Hughes, X Li, T Fu, J Lu, GA McMichael, MA Weiland, MB Eppard, JR Skalski, and RL Townsend

**Supplemental Information**

**JSATS Data Filtering**

The post-processing of JSATS decodes consists of using a sequence of filters to remove false positive decodes and produce acceptable detection events for valid tag codes. False positive decodes are mostly caused by multipath transmissions and mimic decodes. Like other underwater acoustic signals, JSATS transmissions can propagate via direct path as well as multipath (39-40). Multipath transmissions can be from the reflections of JSATS transmissions (at the surface, bottom, and any other structures) or from transmission refraction in the water. Transmission refraction is a consequence of sound speed variation with the depth depending on the temperature, salinity, and pressure. In the shallow river environment that JSATS transmitters are used, transmission refraction is negligible due to the low amount of sound speed variation in the water column, and multipath transmissions are mainly from the reflections of JSATS transmissions.

Another form of false positives comes from what are called mimic decodes, which are the result of a real JSATS transmission being incorrectly decoded. Mimic decodes are the results of the noisy environment that JSATS cabled systems are typically deployed. A JSATS tag code includes a total of 31 bits, with 7 synchronization bits (72 in hexadecimal format), 16 tag identification (ID) bits (4 hexadecimal digits), and 8 cyclic redundancy check (CRC) bits (2 hexadecimal digits). For example, in the tag code 727AACFD, 72 is the Barker code, 7AAC is the tag ID, and FD is the CRC. The JSATS decoder converts a transmission into clipped phase bits to locate the Barker code and decode the following bits using the binary phase shift keying (BPSK) method (23-24). In a noisy field environment, one bit of a tag ID could be distorted, which could then cause the decoder to miss a phase shift or to add one extra bit shifting. In either case, the phases of the distorted bit and the following bits would be inverted (from ‘0’ to ‘1’ or from ‘1’ to ‘0’). Since JSATS transmissions use a CRC, decodes from a distorted transmission are usually not valid tag codes and are discarded. However in some cases a distorted transmission can be decoded as a valid tag code. The decodes resulting from distorted transmissions are called mimic decodes. By simulating the effect of one or multiple inverted bits for each possible tag code a list of known mimic codes can be generated. The filtering method described in the following sections was applied to 2012 JSTAS field study results at John Day Dam (JDA) and The Dalles Dam (TDA).

*Study Sites*

In 2012, the lower Columbia River JSATS field study consisted of 2 seasons: spring season and summer season. During the spring season, 11687 fish were released from 4/23/2012 to 6/2/2012; during the summer season, 14163 fish were release from 6/13/2012 to 7/8/2012. Each fish was surgically implanted with one passive integrated transponder (PIT) tag and one JSATS acoustic transmitter in its body cavity. Tagged fish were released at 5 different fish release sites:

• Release site 1 (R1) is located upstream of the McNary dam (MCN) at river kilometer (rkm) 503 upstream from the mouth of the Columbia River;

• Release site 2 (R2) and release site 3 (R3) are located in between MCN and John Day dam (JDA) at rkm 468 and rkm 422;

• Release site 4 (R4) and release site 5 (R5) are located in between JDA and TDA at rkm 346 and rkm 325.

JSATS cabled arrays were deployed on the dam face of McNary Dam, John Day Dam, The Dalles Dam, and Bonneville Dam on the Lower Columbia River. McNary Dam is located at rkm 470 and includes a navigation lock, powerhouse, spillway, and two adult fish ladders. The powerhouse has 14 turbine units and the spillway has 22 spillway bays. John Day Dam is located at rkm 348 and includes a navigation lock, powerhouse, spillway, and two adult fish passage facilities on both ends of the dam. John Day Dam has 16 turbine units, 4 turbine skeleton bays in-between the powerhouse and the spillway, 20 spillway bays, and a navigation lock. The Dalles Dam is located at rkm 309 and includes a spillway perpendicular to the main river channel with 23 spillway bays, a powerhouse parallel to the main river channel with 22 turbine units on the Oregon side of the river, a navigation lock next to the spillway bays on the Washington shoreline, and two adult fish facilities with one near the spillway and the other one near the powerhouse. Bonneville Dam is located at rkm 234 and includes two powerhouses (B1 and B2), one spillway, two navigation locks, and adult fish passage facilities on both the Washington and Oregon shorelines. JSATS cabled hydrophones were deployed on the dam face using trolleys that are lowered through pipes attached to the dam face. Typically two hydrophones are deployed on each pier nose at the powerhouse and the spillway with one hydrophone at a shallow elevation and the other at a deep elevation. A total of 89 hydrophones were deployed at MCN, 86 at JDA, 79 at TDA, and 85 at BON. In addition, autonomous receiver arrays and PIT detectors were deployed along the Lower Columbia River. Autonomous receiver arrays were deployed at 10 different locations. At each location, a set of receivers were deployed across the river as a detection array using methods described by McMichael et al. (3) and Titzler et al. (22). PIT detectors were installed in juvenile bypass facilities (JBF) at the MCN, JDA, and BON B2 powerhouses. A submerged screen was installed in the turbine intake to guide fish away from turbines (41). When fish passed the cabled array and entered into turbine intake, the screen can guide fish up through channels in the dam and route them into the JBF where the PIT tags will be detected.

*Overall filtering steps*

A sequence of filters were developed to remove false positive decodes and find valid decodes for JSATS cabled arrays (Figure S1). Decodes from each hydrophone were first processed using the multipath filter to remove multipath decodes. Decodes from all hydrophones were then combined together and transmissions that were decoded only by one hydrophone were removed using the single decode filter. A ping rate interval (PRI) filter was then applied to find decodes that exhibit the expected transmission pattern to form detection events (i.e., a group of transmissions within a window) for each tag code. Detection events were then checked using the mimic decode filter to remove mimic events and mimic decodes from 4 known types mimic tag codes. The remaining decodes after applying the mimic decode filter were then processed using the PRI filter again and only decodes and events that passed PRI filter are valid for further analysis.


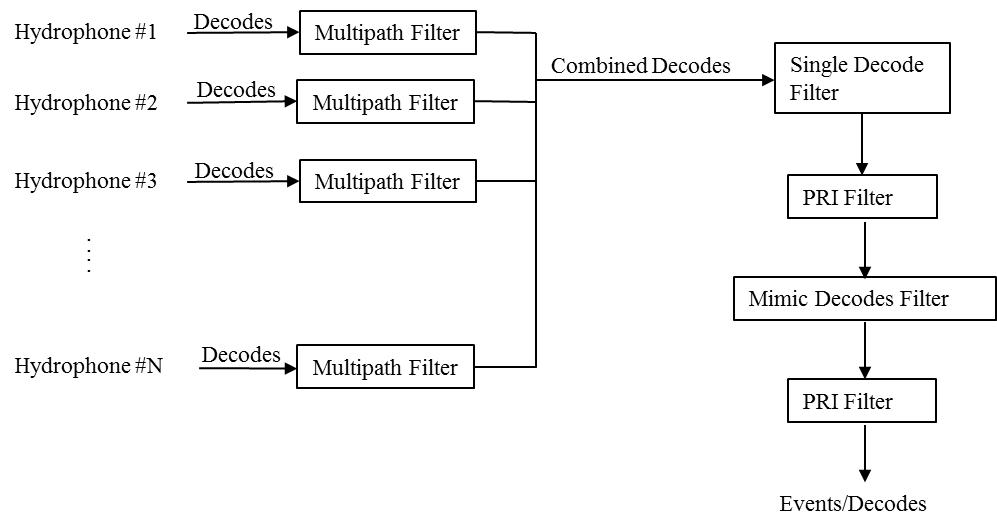


**Figure S1.** Overall filtering steps for JSATS data

*Multipath filter*

The multipath filter checks decodes from each hydrophone for each tag code and removes the decodes resulting from multipath signals. It loops through all decodes from the same hydrophone for a specified tag code using a multipath time window. Since multipath decodes are the reflections of a JSATS transmission from the water surface, river bottom, or other structures, it arrives at a hydrophone later than the same transmission via direct path. Decodes that occur after an initial decode of the same tag code within the time window are multipath decodes and are removed. Only the initial decodes, which are from direct transmission paths, are retained. For the lower Columbia River JSATS cabled array systems, a value of 0.3 s was used as the multipath filter time window.

*Single decode filter*

The single decode filter removes tag transmissions that are only received on one hydrophone within a time window. The single decode filter is applied to the combined decodes from all cabled array hydrophones. It checks the decodes of each individual transmission from a tag code. If there are multiple decodes from different hydrophones for the same tag code within a time window, then all these decodes can pass the filter; if there is only one decode of the tag code in a time window, i.e. a tag transmission was only detected and decoded by one hydrophone, then it is a false positive decode and is removed. In the lower Columbia River field study, the time window was set to 0.3 s representing the maximum hydrophone detection range of 450 m.

*PRI filter*

The PRI filter is designed to use the expected pattern of transmissions to filter out false positive decodes. For JSATS cabled arrays, it checks a set of tag messages within a pre-defined time window and it uses a PRI criteria to find valid messages. A message includes a set of decodes of the same tag transmission from multiple hydrophones. The message time is defined as the earliest time-of-arrival (TOA) of the tag transmission across all hydrophones. The time window is based on a multiple of PRI value and is defined as the nominal PRI*1.3*12+1 seconds. In order for a set of messages to pass the PRI filter, there should be at least N_PRI_ valid messages that match the PRI pattern within the time window. The required N_PRI_ (typically 4, 5, 6, or 7) depends on the acoustic environment.

Starting from the 1st message, the PRI filter finds the number of messages (N_m_) that starts with this initial message and is within the time window. If N_m_ < N_PRI_, then the initial message is invalid and the filter moves to the next message; otherwise, the time difference between the initial message and each following message is calculated and an estimated PRI is calculated for each message. The real PRI of these N_m_ messages is the mode of the estimated PRIs. Each estimated PRI is then compared with the real PRI and only messages that have a difference less than 0.006 seconds are valid messages. If there are more than N_PRI_ valid messages in the time window, then all these valid messages pass the PRI filter and the PRI filter moves to the next message. After applying the PRI filter, continuous messages are formed into a detection event. Each detection event contains at least N_PRI_ messages and represents a continuous or nearly continuous sequence of transmissions.

*Mimic decode filter*

The mimic decode filter is designed to filter out four different known types of mimic decodes:

a. The last 6 tag ID bits and 8 CRC bits are inverted. Due to how the CRC code is calculated, the new CRC is still valid and the decoded code becomes a valid tag code.

b. A tag code has the bit pattern corresponding to the Barker code 72 (1110010) or its inverse (0001101) in the code ID. In this case, the bit pattern corresponding to 72 in the code ID is decoded as Barker code instead of a part of code ID and the following 16 bits are decoded as a code ID. A valid CRC can be added randomly to the signal to form a valid tag code.

c. The last bit of the code ID is inverted which can result in a CRC that is still valid for some tag codes.

d. A tag code that has the last 14 bits of the 16 ID bits inverted, with the original CRC being correctly decoded.

The mimic decodes filter first checks each detection event of a tag code. If a detection event overlaps with another detection event from one of the four possible mimic codes, then the filter compares the messages and decodes for these two overlapping detection events. A decode is a mimic decode if it occurs within a time window of 0.1s of the suspected true decode from the same hydrophone. A message is a mimic message if all decodes of the message are mimic decodes. After all messages in a detection event have been checked and mimic messages have been removed, the PRI filter is applied again to the remaining messages in the detection event. If the detection event can still pass the PRI filter, then it is a valid detection event; otherwise, it is a mimic detection event and is removed.

*Detection Probability*

The detection probability of a detection array (primary array) can be calculated using a group of fish that were detected by a detection array located downstream of this array (secondary array). Using 0 as not detected and 1 as detected, the possible detection histories for each individual fish can be defined as follows:

• 00 : never detected by both arrays

• 10 : detected by the primary array, but not by the secondary array

• 01 : detected by the second array, but not by the primary array

• 11 : detected by both arrays

Assume that fish survived to the secondary array and were detected by the second array, which equals n_01_ + n_11_, representing a random sample of all fish from the group that were alive as they passed the primary array, the detection probability of the primary array is then the proportion of the sample that was detected by the primary array [n_11_ / (n_01_ + n_11_)] (42).

*False Positive Probability*

The false positive probability of a detection array refers to the tags that were never within the detection range, but had false positive detections on the array. For a sample group with *N_s_* tags, the false positive probability of the detection array *P_fp_* can be calculated as: *P_fp_* = *N_fp_*/*N_s_**100%, where *N_fp_* is the number of tags in the sampling group that have false positive detections on the array.

*PRI filter parameter N_PRI_ selection*

In order to find the optimized *N_PRI_* for the JSATS study, values 4, 5, 6, a 7 were chosen as candidates and the data set collected at JDA in the spring was used as the test data set. The goal of the optimization is to find the *N_PRI_* that can maximize the detection probability and minimize the false positive rate of the cabled array. Three groups of fish, which were known to have passed JDA during the spring season, were used to calibrate the detection probability: fish detected by PIT detectors at JDA, fish detected by the autonomous array at the tailrace of JDA, and fish that had three-dimensional tracks in the forebay of JDA. Three dimensional (3D) tracking used decodes of released tags after applying the multipath filter as input. It was used for route-specific survival and required that at least four hydrophones detected the same tag transmission (23). Each track of a released fish was verified manually. For each fish group, the detection probability was calculated after the filtering steps when using *N_PRI_* values of 4, 5, 6, and 7.

The false positive probability was calibrated with 4 groups of fish which were not supposed to be detected by the cabled array at JDA: fish detected by the cabled array at JDA before their release time, fish detected by both cabled array and PIT detectors at JDA after their detection times on PIT detectors, fish released downstream of JDA in the spring season, and fish released downstream of JDA in the summer season. For fish detected by the cabled array at JDA, the first detection time of each fish was compared with its fish release times and fish detected before their release times were false positives. For the fish detected by both the cabled array and PIT detectors at JDA, fish that had detections on cabled array after their PIT detection times were false positives. For fish released downstream of JDA in the spring season and the summer season, fish that passed the filtering steps within these 2 groups were also false positives.

*PRI Filter Calibration*

During the 2012 spring season, 7594 fish were released at three release sites upstream of JDA. Among them, 1349 fish were detected by JDA PIT detectors. When using this group of fish as a reference (G1 in Table S1), the detection probability of the cabled array was the same 99.56% for all four different *N_PRI_* values. For the fish group that were detected by the autonomous receiver array in the tailrace of JDA (G2 in Table S2), the detection probability of cabled array was 99.94% for an *N_PRI_* value of 4, and 99.92% when *N_PRI_* was 5, 6, or 7. For the fish group that had valid 3D tracks (G3 in Table S1), the detection probability of the cabled array was 100.00% when *N_PRI_* was 4, 5, or 6, and when it was 7, one tag was filtered out and the detection probability was 99.98%.

Table S1: Detection probabilities of the JDA cabled array for the different reference tag groups G1, G2, and G3, when different PRI filter parameters (*N_PRI_*) were used. N_01_ is the number of tags that were detected by the downstream array (secondary array), but were not detected by the cabled array at JDA (primary array); N_11_ is the number of tags that were detected by both the cabled array at JDA and the downstream autonomous array.

| Tag Group |  | *N_PRI_* = 4 | *N_PRI_* = 5 | *N_PRI_* = 6 | *N_PRI_* = 7 |
| --- | --- | --- | --- | --- | --- |
|  | N_01_ | 6 | 6 | 6 | 6 |
| G1 | N_11_ | 1343 | 1343 | 1343 | 1343 |
|  | N_01_+N_11_ | 1349 | 1349 | 1349 | 1349 |
|  | Detection Probability | 99.56% | 99.56% | 99.56% | 99.56% |
|  | N_01_ | 4 | 5 | 5 | 5 |
| G2 | N_11_ | 6506 | 6505 | 6505 | 6505 |
|  | N_01_+N_11_ | 6510 | 6510 | 6510 | 6510 |
|  | Detection Probability | 99.94% | 99.92% | 99.92% | 99.92% |
|  | N_01_ | 0 | 0 | 0 | 1 |
| G3 | N_11_ | 6663 | 6663 | 6663 | 6662 |
|  | N_01_+N_11_ | 6663 | 6663 | 6663 | 6663 |
|  | Detection Probability | 100.00% | 100.00% | 100.00% | 99.98% |

**Table S2: False positive probability of the JDA cabled array for the different reference tag groups G1, G2, G3, and G4, when different PRI filter parameters (*N_PRI_*) were used. N_s_ is the number of tags of the reference group. N_fp_ is the number of tags that had false positive detections at JDA cabled array after filtering steps.**

| Tag Group |  | *N_PRI_* = 4 | *N_PRI_* = 5 | *N_PRI_* = 6 | *N_PRI_* = 7 |
| --- | --- | --- | --- | --- | --- |
|  | N_s_ | 6678 | 6677 | 6677 | 6674 |
| G1 | N_fp_ | 30 | 11 | 5 | 0 |
|  | False Positive Probability | 0.45% | 0.16% | 0.07% | 0.00% |
|  | N_s_ | 1343 | 1343 | 1343 | 1343 |
| G2 | N_fp_ | 8 | 5 | 3 | 0 |
|  | False Positive Probability | 0.60% | 0.37% | 0.22% | 0.00% |
|  | N_s_ | 3992 | 3992 | 3992 | 3992 |
| G3 | N_fp_ | 12 | 8 | 4 | 3 |
|  | False Positive Probability | 0.30% | 0.20% | 0.10% | 0.08% |
|  | N_s_ | 7663 | 7663 | 7663 | 7663 |
| G4 | N_fp_ | 7 | 2 | 1 | 1 |
|  | False Positive Probability | 0.09% | 0.03% | 0.01% | 0.01% |

In the spring season, the number of fish that were detected by the cabled array and passed the filtering steps were 6678, 6677, 6677, and 6674 when *N_PRI_* was 4, 5, 6, or 7, respectively. Using fish detected by the cabled array before their release time as the reference group (G1 in Table S2), no fish was detected before its release time and the false positive probability was 0.00% when *N_PRI_* was 7. When *N_PRI_* was 4, 5, or 6 there were 30, 11, and 5 fish respectively that were detected and passed the filtering step, corresponding to false positive probabilities of 0.45%, 0.16%, 0.07% respectively. Using fish detected by both the cabled array and PIT detectors at JDA as the reference tag group (G2 in Table S2), the false positive probability of the JDA cabled array was 0.00% when *N_PRI_* was 7. The probability increased to 0.22%, 0.37%, and 0.60% when *N_PRI_* decreased to 6, 5, and 4. The false positive probability of the fish group released at release sites downstream of JDA during the spring season (3992 fish) were less than 0.1% when *N_PRI_* was 6 or 7, and slightly higher (0.20% and 0.30%) when *N_PRI_* was 4 or 5. For the fish that were released at release sites downstream of JDA Dam in the summer season (7663 tags), the false positive probability was less than 0.1% at *N_PRI_* values of 4, 5, 6, or 7.

The goal of the PRI filter calibration was to find the optimized *N_PRI_* that can maximize the detection probability and minimize the false positive probability. The results show that *N_PRI_* clearly affects the false positive probability (Figure S2): increasing *N_PRI_* can increase the strictness of the PRI filter and reduce the false positive probability. However, different *N_PRI_* values don’t affect the detection probability as strongly as the false positive probability (Table S1): the detection probability was the same for the fish group detected by PIT detectors at JDA at all tested *N_PRI_* values and the difference was only 0.02% (1 tag) for both the fish group detected by the Autonomous array at the tailrace of JDA and the fish group with valid 3D tracks in the forebay of JDA. Thus, the selection of *N_PRI_* was based on the false positive probability with the consideration of the detection probability. Among all tested *N_PRI_* values a value of 7 gave the lowest false positive probability: 0 for two reference groups (G1 and G2) and less than 0.1% for the other two reference tag groups (G3 and G4). For the fish group with valid 3D tracks, the detection probability was 99.98% (missed 1 fish) with an *N_PRI_* of 7. This fish was clearly tracked in the forebay of JDA, and missing it would affect the final dam passage survival. In comparison, the detection results when *N_PRI_* was 6 contained the missed fish. The false positive probabilities of reference fish groups when *N_PRI_* was 6 were very close to when *N_PRI_* was 7: the difference was less than 0.1% for G1, G3, and G4 and 0.22% for G2. Compared with a value of *N_PRI_* of 5, a value of 6 was also a better choice. The filtering results when *N_PRI_* was 5 or 6 had the same detection probabilities for all three reference fish groups, but the false positive probability was lower when *N_PRI_* was 6 for all four reference fish groups. Clearly, an *N_PRI_* of 4 was not a good choice: the false positive probability when *N_PRI_* was 4 was much higher for all reference fish groups than compared to values of 5, 6, or 7. For the purpose of balancing false positive and detection probabilities, an *N_PRI_* value of 6 was chosen as the PRI filter parameter and was used in both the spring summer seasons at JDA and TDA.


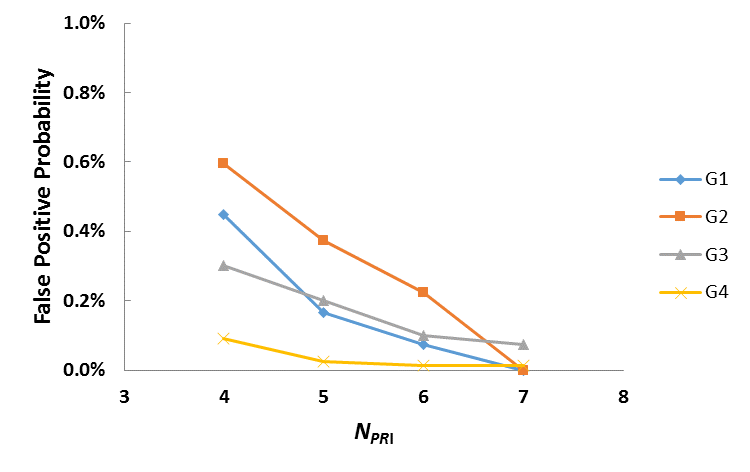


Figure S2: False positive probability of cabled array at JDA for the different reference fish groups when different *N_PRI_* values were used.

*Field Detection Probability*

Field Detection probability was computed using three reference fish groups. The 1^st^ group consists of fish detected by PIT detectors. For the cabled array at JDA, this group included fish detected by PIT detectors at JDA; for the cabled array at TDA, this group included fish detected by PIT detectors at BON B2 powerhouse since there are no PIT detectors at TDA. The 2^nd^ group consists of fish detected by the autonomous receiver arrays downstream of the dam, which included fish detected by the autonomous receiver array in the tailraces of JDA (for cabled array at JDA) and TDA (for cabled array at TDA). The 3^rd^ group consisted of fish that had 3D tracks using cabled array decodes after applying the multipath filter. The detection probability of the 3^rd^ reference group was not computed in the Spring Season at TDA because 3D tracking was not performed for this data set.

In the summer season, the detection probability was 100% for the fish group detected by PIT detectors (G1 in Table S3) at both JDA and TDA. In the spring season, one fish in this group was not detected by the cabled array at TDA and 6 fish in this group were not detected by cabled array at JDA. The detection probability was 99.56% at JDA and 99.89% at TDA. For the fish groups that were detected by the autonomous arrays in the tailraces of JDA and TDA (G2 in Table S3), the detection probability was above 99.9% for all groups. A total of 5 fish in the spring season and 1 fish in the summer season were not detected by the cabled array at JDA. A total of 6 fish in the spring season and 4 fish in the summer season were not detected by cabled array at TDA. For the three fish groups that had 3D tracks (G3 in Table S3), they all passed the filtering steps and had detection probabilities of 100% on the cabled arrays.

Overall, the detection probability was close to or above 99.9% for all reference fish groups except the fish group detected by PIT detectors at JDA in the spring season. When checking the detection histories of these 6 fish on all detection arrays (4 cabled arrays and 10 autonomous receiver arrays), 5 fish were not detected by any cabled arrays or autonomous receiver arrays downstream of JDA. Since each fish was surgically implanted with one JSATS transmitter and one PIT tag, it is very likely that PIT tags in these 5 fish were working properly when they passed JDA, but JSATS transmitters were dropped or not working before they passed JDA. If these 5 fish were excluded, then only one fish in this group was not detected by the cabled array at JDA in the spring season, and the detection probability would increase to 99.93%.

In addition to transmitter issues, fish can pass the dam without being detected by the cabled array if they were never in the detection range of the array. At TDA, the navigation lock is located next to the spillway on the Washington side of the river. The direct distance between the navigation lock and the closest hydrophone was more than 210 m. In the development stage of JSATS, decoding efficiency of the cabled array hydrophones were tested at Bonneville Spillway by placing JSATS transmitters at different distances to the hydrophones (24). The results showed that cabled array hydrophones can detect and decode more than 96% of transmissions when transmitters were within the distance of 72m to the hydrophones. The decoding rate dropped sharply when transmitters were 100 m away from the hydrophones (64.5% at 107 m and less than 20% for one tested hydrophones at 122m). If a fish stayed close to the Washington shoreline at TDA and passed through the navigation lock, they would never be detected by any cabled array hydrophones. At JDA, the navigation lock is 80 m away from the closest hydrophone, but the navigation lock wall sticks out about 200 m from the dam face. The distance between the navigation lock wall and Washington shoreline is about 350 m. If fish were swimming in this area and passed through the navigation lock, transmissions from their transmitters would be blocked by navigation lock wall and wouldn’t be detected by any of the cabled hydrophones. However, any fish that passed through navigation locks would be detected by the autonomous receiver arrays in the tailrace of both JDA and TDA.

Table S3: Detection probability of cabled array at JDA and TDA in 2012 field season for difference reference tag groups G1, G2, G3, and G4. N01 is the number of tags that were detected by the downstream array (secondary array), but were not detected by the cabled array (primary array); N11 is the number of tags that were detected by both cabled array and downstream array.

|  |  | JDA | | TDA | |
| --- | --- | --- | --- | --- | --- |
| Tag Group |  | Spring | Summer | Spring | Summer |
|  | N_01_ | 6 | 0 | 1 | 0 |
| G1 | N_11_ | 1343 | 937 | 937 | 324 |
|  | N_01_+N_11_ | 1349 | 937 | 938 | 324 |
|  | Detection Probability | 99.56% | 100.00% | 99.89% | 100.00% |
|  | N_01_ | 5 | 1 | 6 | 4 |
| G2 | N_11_ | 6505 | 6540 | 9785 | 7007 |
|  | N_01_+N_11_ | 6510 | 6541 | 9791 | 7011 |
|  | Detection Probability | 99.92% | 99.98% | 99.94% | 99.94% |
|  | N_01_ | 0 | 0 | NA | 0 |
| G3 | N_11_ | 6663 | 5767 | NA | 7301 |
|  | N_01_+N_11_ | 6663 | 5767 | NA | 7301 |
|  | Detection Probability | 100.00% | 100.00% | NA | 100.00% |

*Field False Positive Probability*

The field false positive probability was computed using three reference fish groups: the 1^st^ reference group consisted of fish detected by the cabled array at JDA and TDA before their release time (G1 in Table S4); the 2^nd^ reference group consisted of fish detected both by the cabled arrays and the PIT detectors (PIT detectors at JDA for the JDA cabled array and PIT detectors at BON B2 powerhouse for the TDA cabled array; G2 in Table S4). Fish detected by the cabled array after their detection times at PIT detectors were false positives. The last reference group consisted of fish detected by both the cabled arrays and the autonomous receiver arrays at the tailrace of JDA and TDA (G3 in Table S4). Fish detected by the cabled array before their detection times at autonomous receiver arrays were false positives.

The false positive probability was less than 0.23% for all reference fish groups. During the summer season, the false positive probability was 0.00% for all three reference fish groups. During the spring season, the false positive probability was 0.03% at both dams for the G1 group. Only 2 fish at JDA and 3 fish at TDA had detections before their release time. The false positive probability of the G2 group was the largest among all three groups during the spring season: 0.22% at JDA and 0.21% at TDA. The false positive probability was less than 0.1% for the G3 fish group: 0.09% at JDA Dam and 0.05% at TDA Dam in the spring season.

Table S4: False positive probabilities of cable array detections at JDA and TDA. N_s_ is the number of tags of the sampling group. N_fp_ is the number of tags that had false positive decodes after filtering steps.

|  |  | JDA | | TDA | |
| --- | --- | --- | --- | --- | --- |
| Tag Group |  | Spring | Summer | Spring | Summer |
|  | N_s_ | 6676 | 5777 | 10307 | 7325 |
| G1 | N_fp_ | 2 | 0 | 3 | 0 |
|  | False Positive Probability | 0.03% | 0.00% | 0.03% | 0.00% |
|  | N_s_ | 1343 | 937 | 937 | 324 |
| G2 | N_fp_ | 3 | 0 | 2 | 0 |
|  | False Positive Probability | 0.22% | 0.00% | 0.21% | 0.00% |
|  | N_s_ | 6505 | 5460 | 9778 | 7007 |
| G2 | N_fp_ | 6 | 0 | 5 | 0 |
|  | False Positive Probability | 0.09% | 0.00% | 0.05% | 0.00% |

**Water Temperature and River Flow**

The river environment experienced by tagged subyearling Chinook salmon of each group was characterized by warmer-than-average water temperatures in both the Snake and Columbia rivers and below-average discharge in the Snake River in 2013. The mean temperature of the Snake River (as measured at LMN) was between 16^o^C and 18^o^C during the first week of the in-river survival comparison study period, which was about 0.5^o^C warmer than the ten-year average for this time of year (Figure S3). However, the river warmed quickly in 2013 beginning around 9 July, with the mean daily temperature reaching 20^o^C by 10 July, which was about 1^o^C warmer than the ten-year average. The Snake River remained warmer than average through July and the mean daily temperature remained between 20^o^C and 21^o^C from 12 July through the end of the study.


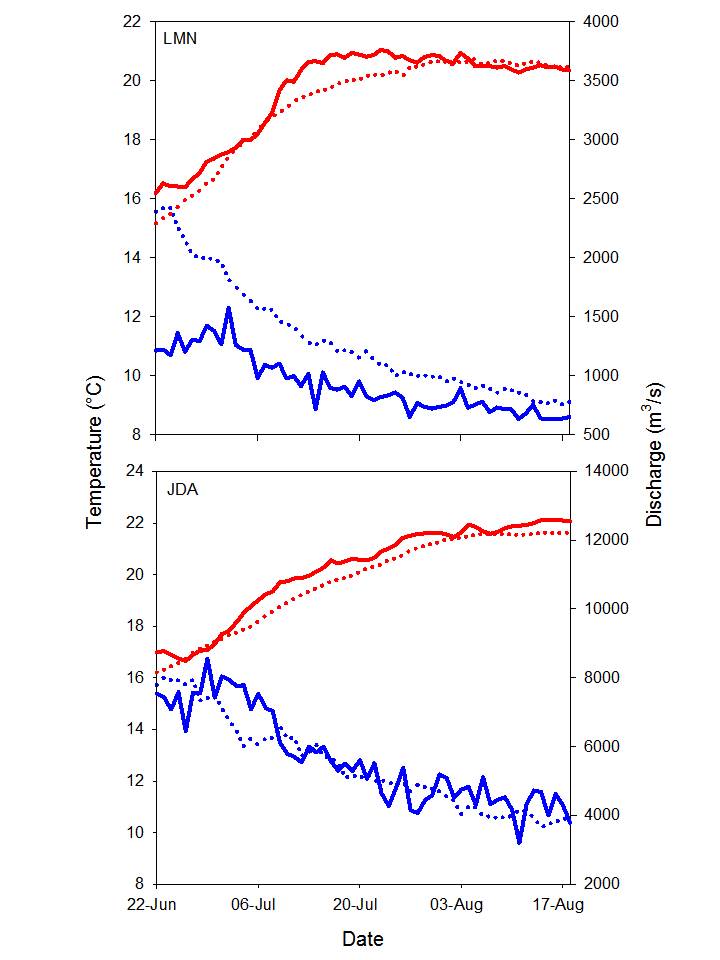


**Figure S3.** Water temperature (^o^C; red) and discharge (m^3^/s; blue) as measured at Lower Monumental Dam (LMN; top panel) and John Day Dam (JDA; bottom panel) during the 2013 in-river survival comparison (solid lines) relative to the ten-year (2003–2012) average (dotted lines). Temperature and discharge data is from Columbia River DART (<http://www.cbr.washington.edu/dart>).

The warm water temperatures experienced by summer migrants in the Snake River were accompanied by lower-than-average flows. At LMN, discharge declined steadily throughout the study period, which followed the trend of the ten-year average. However, in 2013, discharge was well below the ten-year average throughout the entire study period. During the first two weeks, discharge at LMN ranged from about 1150 m^3^/s to 1600 m^3^/s, which was about 250 m^3^/s to 1250 m^3^/s lower than the ten-year average. By late July, flows in the Snake River began approaching the ten-year average; however, most tagged fish had exited the Snake River by this time.

The mean daily temperature in the Columbia River (as measured at John Day Dam; JDA) remained about 0.5^o^C above the ten-year average throughout most of the in-river survival comparison study period in 2013 (Figure S3). The mean daily temperature of the Columbia River at JDA exceeded 20^o^C on 14 July, 21^o^C on 24 July, and 22^o^C on 13 August. Therefore the mean daily temperature experienced by the majority of tagged fish once in the Columbia River ranged from about 20^o^C to 22^o^C. Discharge generally followed the ten-year average in the Columbia River throughout the in-river survival comparison study period.

River temperature may have contributed to the difference in survival observed between fish implanted with the injectable transmitter and those surgically implanted with the single-battery JSATS transmitter. As mentioned, the temperature of the Snake and Columbia rivers was above-average during the subyearling Chinook salmon outmigration period of 2013. The temperature experienced by the majority of tagged fish ranged from about 16^o^C to 20^o^C in the Snake River and from about 20^o^C to 22^o^C in the Columbia River. The chronic upper thermal limit depends on the acclimation temperature, but has generally been found to be within the range of 25^o^C to 27^o^C for juvenile Chinook salmon that were acclimated to temperatures that resembled those experienced by subyearling Chinook salmon in the Snake River during the summer of 2013 (43-44). Although laboratory experiments demonstrated that fall Chinook salmon juveniles can survive and grow at temperatures up to 24^o^C, juveniles reared at 21–24^o^C experienced significant decreases in growth rates, impaired smoltification indices, and increased vulnerability to predation compared to juveniles reared at cooler temperatures (45). Most studies agree that temperatures of about 19^o^C to 20.5^o^C are optimal for Chinook salmon fed to satiation; however, the optimal growth temperature may be considerably lower (i.e., 15^o^C) at feeding levels more common to fish in the wild (60% of satiation) and sublethal growth stress can occur at 18–19^o^C (44). Therefore, it is likely that tagged subyearling Chinook salmon experienced some level of temperature-induced stress during their emigration through the Snake and Columbia rivers in 2013.

Environmental stressors have been found to exacerbate the effects of transmitter attachment (45, 46). Both the immune and inflammatory responses of fish are mediated by temperature (47, 48). Although fish may heal more quickly at warmer temperatures, rates of inflammation and infection are also higher at warmer temperatures. Therefore, the implantation of transmitters may adversely affect the health and behavior of study fish at warm temperatures (48). Bluegills implanted with radio transmitters had higher inflammation and necrosis at incision and suture sites when held at 18^o^C compared to those held at 6^o^C (48). Walsh et al. (46) observed rapid healing of sutured incisions but significant delayed effects of irritation, infection, and mortality in hybrid striped bass implanted with simulated radio tags and held at high temperatures (22–29^o^C). Deters et al. (49) found juvenile Chinook salmon surgically implanted with acoustic transmitters had lower tag and suture retention and higher incision openness, wound inflammation, and ulceration 7 and 14 d postsurgery when held at 17^o^C compared to those held at 12^o^C. The first significant difference in reach survival observed during the current study between the injectable and surgically implanted groups occurred after the fish had already traveled 53 km and had been in the river for about 4 to 7 days postsurgery. Therefore, it is possible the high temperatures led to increased irritation and infection rates in fish surgically implanted with the single-battery JSATS transmitter, which took some time to develop into a condition severe enough to cause mortality.

Additional Figures and Tables

Table S5. Mean, standard deviation (SD), and range of fork lengths (mm) and weights (g) of subyearling Chinook salmon injected with injectable transmitters (Injectable) or surgically implanted with the single-battery JSATS transmitters (Surgical) at Lower Monumental Dam in 2013 for the in-river survival comparison conducted to identify differences in survival between fish implanted with the different transmitter types.

|  |  | Fork Length (mm) | | | Weight (g) | | |
| --- | --- | --- | --- | --- | --- | --- | --- |
|  | *N* | Mean | SD | Range | Mean | SD | Range |
| Injectable | 683 | 109.4 | 7.5 | 95−143 | 12.8 | 2.9 | 7.5–29.3 |
| Surgical | 1033 | 110.6 | 7.7 | 95–145 | 13.8 | 3.3 | 7.4–33.2 |


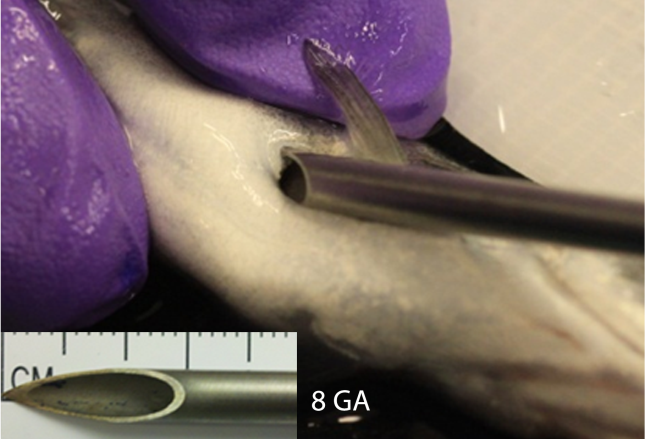


Figure S4. Injectable transmitter implantation in a subyearling Chinook salmon and a photo of the gauge 8 needle used for the implantation.


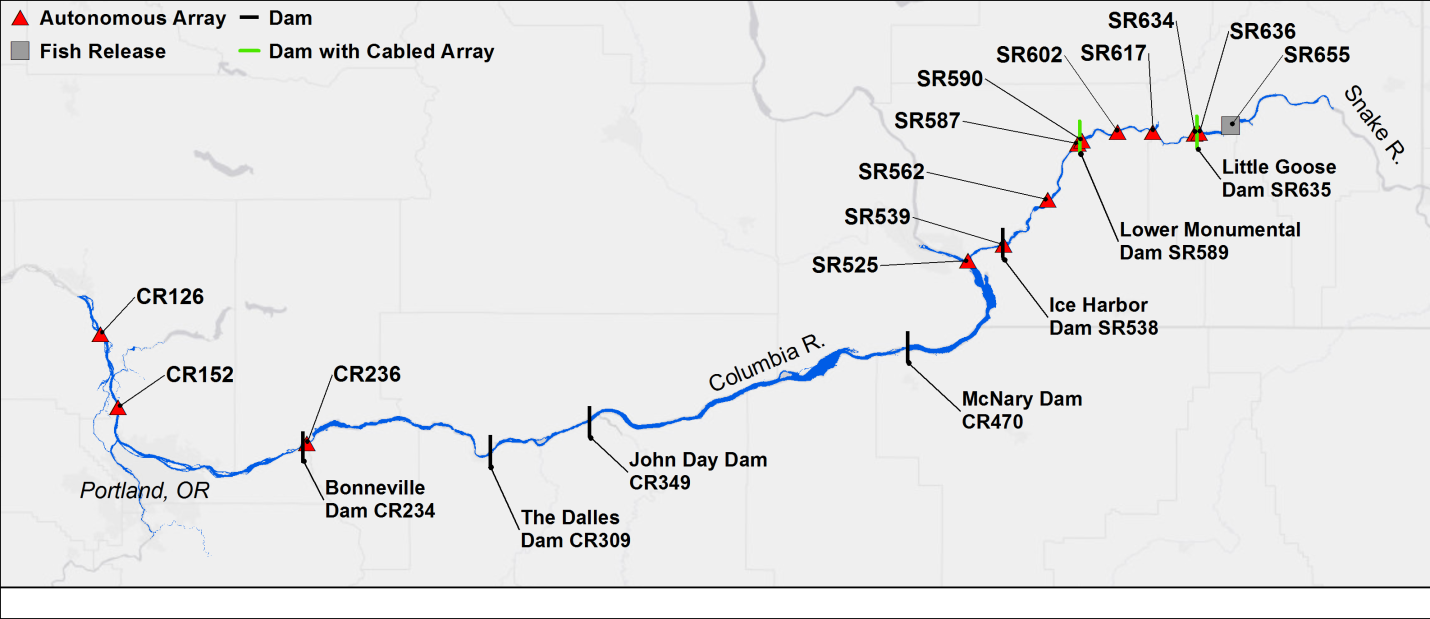


Figure S5. Locations of fish releases, autonomous receiver arrays, and cabled dam-face arrays used to evaluate the performance of the injectable transmitter and the survival of fish implanted with the injectable transmitter. Black location codes (e.g., SR133) give the river kilometer referenced from the mouth of the Snake River, while red location codes (e.g., RKM655) give the river kilometer from the mouth of the Columbia River. Map Generated using ArcGIS 10.3 (www.arcgis.com).

Table S6. Locations and types of acoustic receiver detection arrays. River kilometers are given from the mouth of the Columbia River.

| Array Name | River Kilometer (m) | Array Type | # of Nodes |
| --- | --- | --- | --- |
| Little Goose Dam Forebay | 636 | Autonomous | 4 |
| Little Goose Dam | 635 | Cabled | 33 |
| Little Goose Dam Tailrace | 634 | Autonomous | 3 |
| Lyons Ferry State Park, WA | 617 | Autonomous | 6 |
| Ayer’s Boat Basin, WA | 602 | Autonomous | 4 |
| Lower Monumental Dam Forebay | 590 | Autonomous | 4 |
| Lower Monumental Dam | 589 | Cabled | 38 |
| Lower Monumental Dam Tailrace | 587 | Autonomous | 3 |
| Snake River Road Launch | 562 | Autonomous | 4 |
| Ice Harbor Dam Forebay | 539 | Autonomous | 4 |
| Burbank, WA | 525 | Autonomous | 4 |
| Bonneville Dam Forebay | 236 | Autonomous | 4 |
| Knapp, WA | 152 | Autonomous | 8 |
| Kalama, WA | 126 | Autonomous | 8 |

**Table S7.** Reach-specific survival estimates and associated standard errors (SE) for fish implanted with the injectable transmitter compared to those of the control group, which were surgically implanted with the single-battery JSATS transmitter. The sample sizes (*N*) of fish detected by the upstream array of each reach that were used to estimate the single-release reach-specific survival probabilities are displayed, as are the results (*χ*^2^ and *P*) of likelihood ratio tests used to identify statistically significant (*P* < 0.05; indicated by *) differences in survival between the two groups.

|  |  | Injectable | | Single-battery (surgical) | |  |  |
| --- | --- | --- | --- | --- | --- | --- | --- |
| From (rkm) | To (rkm) | *N* | *S* (SE) | *N* | *S* (SE) | *χ*^2^ | *P* |
| Release (rkm 655) | 636 | 683 | 0.866 (0.013) | 1033 | 0.856 (0.011) | 0.371 | 0.543 |
| 636 | 635 | 588 | 0.993 (0.003) | 882 | 0.992 (0.003) | 0.062 | 0.804 |
| 635 | 617 | 585 | 0.891 (0.013) | 876 | 0.874 (0.011) | 0.882 | 0.348 |
| 617 | 602 | 521 | 0.927 (0.011) | 767 | 0.927 (0.009) | 0.000 | 0.996 |
| 602 | 590 | 483 | 0.915 (0.013) | 711 | 0.861 (0.013) | 8.501 | 0.004* |
| 590 | 589 | 442 | 0.984 (0.006) | 612 | 0.982 (0.005) | 0.070 | 0.791 |
| 589 | 562 | 435 | 0.903 (0.014) | 601 | 0.845 (0.015) | 7.758 | 0.005* |
| 562 | 539 | 393 | 0.875 (0.017) | 508 | 0.902 (0.013) | 1.551 | 0.213 |
| 539 | 525 | 344 | 0.933 (0.014) | 458 | 0.928 (0.012) | 0.086 | 0.770 |
| 525 | 236 | 321 | 0.601 (0.027) | 423 | 0.523 (0.024) | 4.603 | 0.032* |
| 236 | 152 | 193 | 0.928 (0.019) | 222 | 0.928 (0.017) | 0.000 | 0.993 |
